# Chronic Voluntary Ethanol Consumption Induces Favorable Ceramide Profiles in Selectively Bred Alcohol-Preferring (P) Rats

**DOI:** 10.1371/journal.pone.0139012

**Published:** 2015-09-25

**Authors:** Jessica Godfrey, Lisa Jeanguenin, Norma Castro, Jeffrey J. Olney, Jason Dudley, Joseph Pipkin, Stanley M. Walls, Wei Wang, Deron R. Herr, Greg L. Harris, Susan M. Brasser

**Affiliations:** 1 Department of Psychology, San Diego State University, San Diego, California, United States of America; 2 Department of Biology, San Diego State University, San Diego, California, United States of America; 3 Department of Pharmacology, Yong Loo Lin School of Medicine, National University of Singapore, Singapore, Singapore; Universidade do Estado do Rio de Janeiro, BRAZIL

## Abstract

Heavy alcohol consumption has detrimental neurologic effects, inducing widespread neuronal loss in both fetuses and adults. One proposed mechanism of ethanol-induced cell loss with sufficient exposure is an elevation in concentrations of bioactive lipids that mediate apoptosis, including the membrane sphingolipid metabolites ceramide and sphingosine. While these naturally-occurring lipids serve as important modulators of normal neuronal development, elevated levels resulting from various extracellular insults have been implicated in pathological apoptosis of neurons and oligodendrocytes in several neuroinflammatory and neurodegenerative disorders. Prior work has shown that acute administration of ethanol to developing mice increases levels of ceramide in multiple brain regions, hypothesized to be a mediator of fetal alcohol-induced neuronal loss. Elevated ceramide levels have also been implicated in ethanol-mediated neurodegeneration in adult animals and humans. Here, we determined the effect of chronic voluntary ethanol consumption on lipid profiles in brain and peripheral tissues from adult alcohol-preferring (P) rats to further examine alterations in lipid composition as a potential contributor to ethanol-induced cellular damage. P rats were exposed for 13 weeks to a 20% ethanol intermittent-access drinking paradigm (45 ethanol sessions total) or were given access only to water (control). Following the final session, tissues were collected for subsequent chromatographic analysis of lipid content and enzymatic gene expression. Contrary to expectations, ethanol-exposed rats displayed substantial reductions in concentrations of ceramides in forebrain and heart relative to non-exposed controls, and modest but significant decreases in liver cholesterol. qRT-PCR analysis showed a reduction in the expression of sphingolipid delta(4)-desaturase (*Degs2*), an enzyme involved in *de novo* ceramide synthesis. These findings indicate that ethanol intake levels achieved by alcohol-preferring P rats as a result of chronic voluntary exposure may have favorable vs. detrimental effects on lipid profiles in this genetic line, consistent with data supporting beneficial cardioprotective and neuroprotective effects of moderate ethanol consumption.

## Introduction

Alcohol abuse has serious consequences on physical and mental health, representing a major public health burden [1–2]. While light-to-moderate drinking has been associated with health benefits such as decreased rates of cardiovascular disease and reduced risks for dementia, type 2 diabetes, and osteoporosis, excessive alcohol intake can cause chronic liver injury/cirrhosis as well as adverse effects on multiple organ systems, leading to gastrointestinal, cardiac, musculoskeletal, immune system and other disorders [3–4]. Heavy alcohol consumption is also well documented to have detrimental neurologic effects, inducing widespread neuronal loss/neurodegeneration in both fetuses and adult organisms in areas such as cerebellum, hippocampus, entorhinal cortex, anterior cingulate, and superior frontal association cortex [5–10], as well as significant white matter atrophy [11].

One of several proposed mechanisms of ethanol-induced cell loss with sufficient exposure is an elevation in concentrations of bioactive lipids that mediate apoptosis, including the sphingolipid metabolites ceramide and sphingosine [12–17]. These latter molecules are components of naturally-occurring sphingomyelin, a class of functional plasma membrane phospholipids found in all eukaryotic cells and ubiquitous in the mammalian nervous system [18]. While sphingolipids such as ceramide are critical physiological modulators of normal neuronal development, differentiation, and apoptosis, elevated levels resulting from various extracellular insults have also been implicated in pathological apoptosis of neurons and oligodendrocytes in several neuroinflammatory and neurodegenerative disorders, including Alzheimer’s disease, HIV-associated dementia, multiple sclerosis, amyotrophic lateral sclerosis, stroke, and aging [15], as well as alcohol-induced central nervous system damage [19–21]. Previous work has demonstrated that a single dose of ethanol administered to pregnant C57BL/6J mice during gestational day (GD) 15–16 results in increased levels of both ceramide and sphingosine in the brains of offspring, hypothesized to be a potential mediator of fetal alcohol-induced neuronal loss [22]. Similarly, acute ethanol administration to mice on postnatal day (PD) 7, equivalent to the late third trimester in humans, elevates levels of ceramide and other lipids in multiple brain regions, including cortex, inferior colliculus, and hippocampus, with corresponding increases in capsase 3 activation, an enzymatic marker of apoptosis [21]. Directly inhibiting the rate-limiting enzyme for ceramide synthesis, serine palmitoyltransferase (SPT), reduced ethanol-mediated increases in both measures, as well as indices of neurodegeneration, strongly implicating ethanol-induced increases in ceramide in its neurodegenerative effects on the developing brain [21]. These findings agree with prior data of the involvement of *de novo* ceramide synthesis in ethanol-induced apoptosis in cultured neurons [23].

Elevated levels of ceramide have also been mechanistically implicated in contributing to ethanol-mediated neurodegeneration in adult animals and humans. In addition to alcohol’s direct toxic effects on the brain, chronic ethanol exposure also induces liver injury/steatohepatitis (i.e., fatty liver disease), accompanied by insulin resistance and increased production of peripheral ceramides, which can freely penetrate the blood-brain barrier to mediate central nervous system insulin resistance and cellular damage [20]. Genetic factors appear to play a role in vulnerability to alcohol-induced hepatic steatosis and associated neurodegeneration, with significant rodent strain differences in these measures [20,24–26], which correlate with liver and blood ceramide concentrations [20]. Chronic alcohol feeding in susceptible rodent strains also results in elevated expression of pro-ceramide genes in the liver, consistent with that found in brains of chronic alcoholics [20]. Further, exogenous in vivo (i.p.) administration of ceramide has been shown to produce neurodegenerative effects and cognitive deficits that parallel chronic ethanol intake [20].

The present study aimed to determine the effect of chronic voluntary ethanol consumption on lipid profiles in brain and peripheral tissues from adult organisms to further examine alterations in lipid composition as a potential contributor to ethanol-induced nervous system and other organ pathology. Toward this aim, we utilized a selectively bred high-drinking rodent line (P) and an established model of chronic ethanol exposure (i.e., 20% ethanol intermittent access paradigm [27–29]), followed by collection of blood, liver, brain, heart, and muscle tissue for subsequent chromatographic analyses of lipid content.

## Materials and Methods

### Animals

Naive adult male selectively bred alcohol-preferring (P) rats (*n =* 21, 68th-69th generations; Indiana University School of Medicine Alcohol Research Center, Indianapolis, IN) were used. This selectively bred line was originally derived from a Wistar foundation stock as described by Lumeng, Hawkins & Li [30]. Rats were 10–16 weeks of age at the start of the experiment with a mean body weight of 409.57 g (±13.31 standard error of the mean (SEM)). During the course of experimental procedures, animals were housed individually in standard modular test chambers (30.5 × 24 × 21 cm) equipped with dual lickometers (Med Associates, Inc., St. Albans, VT) within a testing room maintained on a 12:12 hr light/dark cycle and at an ambient temperature of approximately 23°C. Food and water were available *ad libitum*. Rats were tested in squads of 10 and 11 subjects, with each squad comprised as equally as possible of ethanol-exposed and non-exposed (water only) controls. This study was approved by the Institutional Animal Care and Use Committee at San Diego State University and was in accordance with National Institutes of Health guidelines.

### Chronic ethanol exposure

Non-deprived P rats were exposed for 13 weeks to either a 20% ethanol intermittent access drinking paradigm (*n* = 11) or were given access only to water (*n* = 10). This alcohol exposure paradigm has previously been shown to result in elevated voluntary ethanol drinking in standard outbred rats as well as rats genetically selected for high levels of oral alcohol consumption [27–29]. The first five daily sessions served to acclimate the animals to the apparatus and testing procedures and all rats received free access to food and water only. Following the acclimation period, half of the rats (ethanol-exposed) began 22-hr intake sessions involving voluntary access to a 20% (v/v) ethanol solution vs. water, alternating with 22-hr abstinence periods involving voluntary access to water only (45 ethanol drinking sessions total over 13 weeks). The position of ethanol and water bottles was rotated each ethanol session to control for position preferences. A control group of non-ethanol-exposed rats was given voluntary access to water only during the entire duration of the chronic exposure period (94 sessions). All fluids were weighed to the nearest gram and replaced daily, and body weights were measured every 48 hr. During each intake session, lick activity (raw interlick interval and lick count data) was detected on each tube via an AC contact circuit and recorded precisely by computer and associated software (Med Associates, Inc.) for later quantitative analysis. One day following their final experimental session, rats were deeply anesthetized with sodium pentobarbital, euthanized, and tissues collected for subsequent analysis of lipid content.

### Thin layer chromatography analysis of lipid content

Levels of ceramides, cholesterol, phosphatidylcholine (PC), phosphatidylethanolamine (PE), and sphingosine were measured in forebrain, heart, liver, muscle, and blood. Briefly, 5 mg of each tissue type was mechanically homogenized in 1.0 ml of 2:1 chloroform:methanol. Samples were mix vortexed and centrifuged at 8000 rpm at 4°C and supernatant was transferred to a new glass vial and mix vortexed. High performance thin layer chromatography silica glass plates were pretreated with 1:1 dichloromethane:methanol and dried. 10 ul of each sample was spotted and allowed to dry for 15 minutes. Samples detecting ceramide and sphingosine were run in a solvent containing 9:1 chloroform:methanol. For phospholipids and simple lipids, a solvent containing 65:25:5 chloroform:methanol:water was used. Plates were dried for 1 hour, sprayed with a 10% solution of Copper(II) Sulfate in 10% phosphoric acid, dried an additional 30 minutes, and charred at 125°C for 10 minutes. Lipids were identified by migration distance (Rf) and relative levels quantified by optical density measurements using ImageJ software. Extracts from tissues for each TLC plate were prepared simultaneously, were run side-by-side and comprised equal numbers of samples from each experimental group. Sample identities remained blinded until after completion and analysis of all samples in the study.

### Gene expression analysis

Total RNA was isolated from approximately 50 mg of forebrain tissue using Trizol reagent (Life Technologies) per manufacturer’s instructions. Approximately 2 μg of each sample was primed with oligo(dT)_18_ and random hexamer primers prior using Thermo Scientific Maxima First Strand cDNA synthesis kit (Life Technologies). For quantitative real-time RT-PCR, targets were amplified with Maxima SYBR Green/ROX qPCR Master Mix (Life Technologies) on an Applied Biosystems ViiA 7 Real-Time PCR System (Life Technologies) using gene-specific primer pairs (see below). Relative gene expression was determined using the 2^-ΔΔCT^ method as described [31].

### Primer sets used for gene expression analysis

Gene Direction Sequence

*Sptlc1* forward CAGACCATCCACAAGTCCCT

reverse GTAGCGTGCCTGAGTCAATG

*Cers1* forward TGTGCCTGACATTCCGTACT

reverse TCCAGACTGTCGTATTCCCG

*Cers2* forward CCTCTTCATTGTCTTCGCCG

reverse CAGGGTAGAACTCCAGTGGG

*Cers3* forward TTGTGAAAGCGTCCCACTTG

Reverse ACGGTTGACTTGTGGAATGC

*Cers4* forward CTCATCCTGCGCATGATCTG

reverse GCCATCCCATTCTTCAGCTG

*Cers5* forward CTATCTCACACAGCTGGCCT

reverse CACTCGCACCATGTTGTTGA

*Cers6* forward TTCTGCATCTTCATGGTGCG

reverse GGATGCTTTGTTATGGCGGT

*Smpd1* forward CCTTCACTGGGACCATGACT

reverse ACACTTGCTGTACTCTCCCC

*Smpd2* forward ATGGATCAGCGGAAAGGTCA

reverse GCATTATGGCCACTTCCCTG

*Smpd3* forward TCTTCTGGTCTCCACTGCAG

reverse ATTATTGAGCCTTGCGAGCG

*Asah1* forward CCGTGGACAGAAGATTGCAG

reverse AGTTCTCAACACAGGTGCCT

*Asah2* forward TGTAGGCGCTAACCCAAAGA

reverse TGCATTGCTCAGACCCAGTA

*Acer1* forward TGGTGGCCGAGTTCTACAAT

reverse GGAGATCTCATCCAGCAGCT

*Acer2* forward TGTGACAATGTGCGTGTGTT

reverse GCAAGGCAGATGAGGATGTG

*Acer3* forward TTATCCGTGGCTCAGAGGAC

reverse GCCACCATGCATGAAACTGT

*Sgpl1* forward GTCCTGTGTACTCTGCTGGT

reverse TCTCCAGAACCTCAGCTGTG

*Degs1* forward CTGGTTTGGAATGTTCGCGA

reverse CCCAGACAAGCTTCCTGAGA

*Degs2* forward CCTTCTAGCCAGCTCTCTCC

reverse AATCATCCGTACCAGTGGCA

For gene full names, refer to S1 Appendix.

### Statistical analysis

Ethanol intake and preference (ratio of ethanol consumed to total fluid intake) in ethanol-exposed rats were calculated for each session during the 13-week chronic exposure phase to determine levels and patterns of ethanol consumption across time. Additionally, a microstructural bout analysis was performed on the individual lick latency data from each session to examine alterations in detailed components of ingestive behavior over the course of chronic exposure. Microstructural measures were obtained by analyzing the raw interlick interval data from each session, with a drinking episode or “bout” operationally defined as an occurrence of 20 or more successive licks on a given tube separated by interlick intervals of less than two minutes. For each session, the following measures were calculated for each solution: total # of bouts (bout frequency), mean # licks/bout (bout size), mean bout duration, mean within-bout rate of ingestion, and total # of licks. Individual session data were averaged in 3-session blocks prior to analysis (15 blocks total).

Ethanol and water intake, ethanol preference and microstructural measures in ethanol-exposed subjects were subsequently analyzed using repeated measures analysis of variance (ANOVA) with solution and block as within-subject factors. Overall fluid intake and body weight in ethanol-exposed and control subjects were compared using mixed ANOVAs with chronic exposure treatment (ethanol or control) as a between-subject factor and block or day as within-subject factors. Significant main effects or interactions from the overall ANOVAs were further analyzed using Newman-Keuls test where appropriate. Levels of lipid constituents from each tissue type for ethanol-exposed and control subjects were analyzed using one-way between-subjects treatment ANOVAs on the optical density values and represented as percent of control values. Differences in relative gene expression levels were analyzed by Student’s t-test. Alpha level for all statistical tests was 0.05.

## Results

### Ethanol Intake and Preference–Chronic Exposure Phase

**Fig 1:**
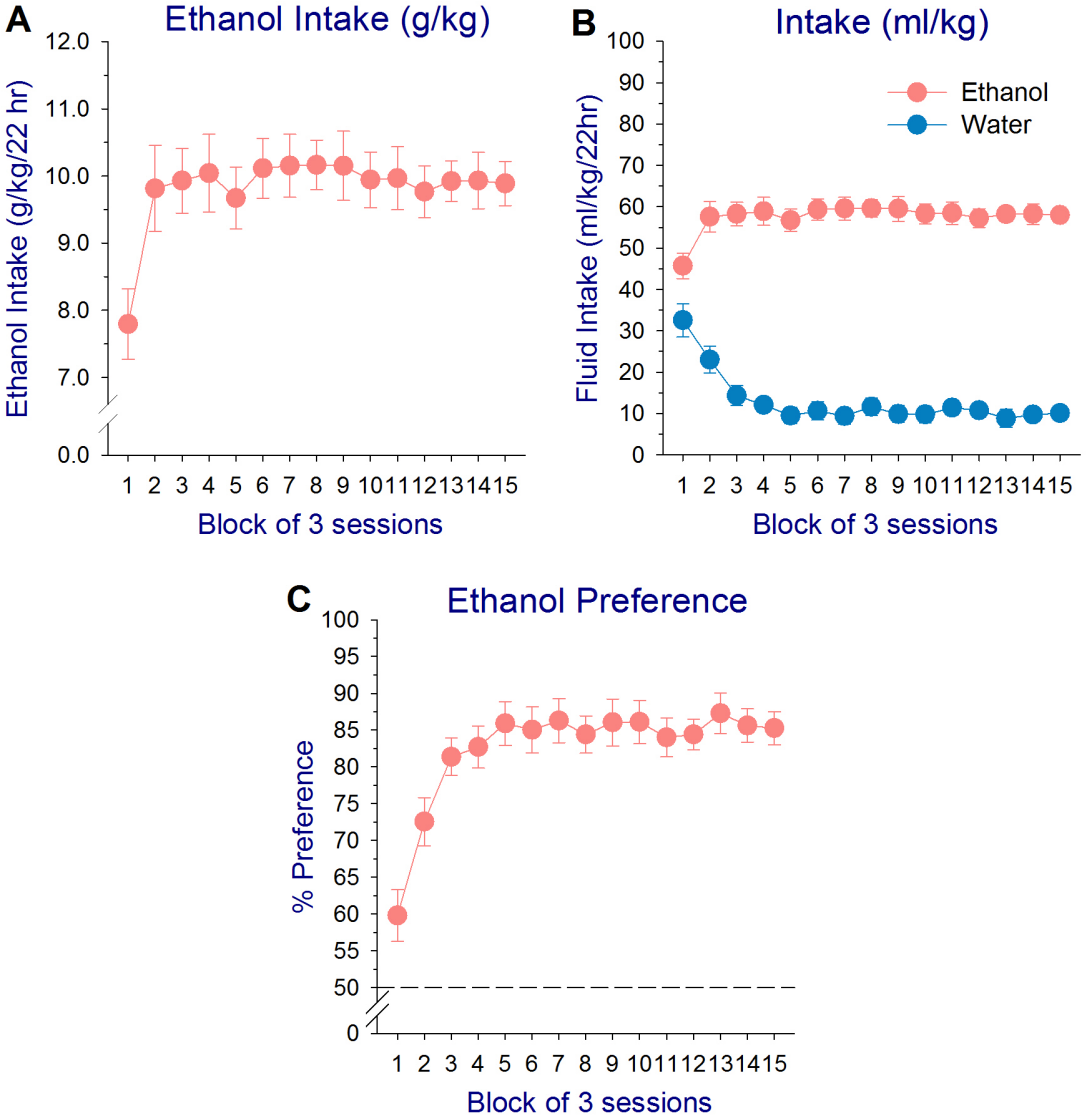
Mean (±SEM) g/kg ethanol intake (A), ml/kg ethanol and water intake (B), and ethanol preference (ratio of ethanol consumed to total fluid intake; C) in selectively bred alcohol-preferring (P) rats across all 15 session blocks of ethanol exposure (45 sessions total) in an intermittent-access 20% ethanol drinking paradigm. Individual session data were averaged in 3-session blocks prior to analysis.

The intermittent-access 20% ethanol drinking paradigm resulted in elevated intake of ethanol by alcohol-preferring (P) rats across initial days of ethanol testing, followed by a stable pattern of ethanol intake during the remainder of the chronic exposure period. A one-way repeated measures ANOVA on g/kg ethanol intake in ethanol-exposed subjects revealed a significant effect of session block (*F*_14,140_ = 4.80, *P* < 0.001), with ethanol intake during block 1 (sessions 1–3) significantly less than all other blocks (Newman-Keuls test: *P*’s < 0.001), while intake across blocks 2–15 did not differ (*P*’s > 0.80; Fig 1A). Analysis of ml/kg ethanol and water intake in ethanol-exposed subjects revealed a similar initial increase in ethanol intake, while water intake declined (main effect of solution: *F*_1,10_ = 176.63, *P* < 0.001; main effect of block: *F*_14,140_ = 3.47, *P* < 0.001; solution × block interaction: *F*_14,140_ = 18.57, *P* < 0.001; Newman-Keuls test: ethanol intake–block 1< 2–15; water intake–block 1>2–15, block 2>3–15, *P*’s < 0.001). Ml/kg ethanol intake was significantly greater than water intake for all blocks (Newman-Keuls test, *P*’s < 0.001; Fig 1B). A two-way repeated measures ANOVA on the total lick counts/session for ethanol and water in ethanol-exposed animals also confirmed similar patterns of intake as the fluid intake measures (main effect of solution: *F*_1,10_ = 101.03, *P* < 0.001); solution × block interaction: *F*_14,140_ = 4.52, *P* < 0.001). Mean lick counts/session for 20% ethanol by P rats increased across blocks 1–3 and then stabilized, while lick counts to water displayed a parallel decrease (Newman-Keuls test: ethanol licks–block 1< 3–8, 12–15; water licks–block 1>3–15, block 2>11, *P*’s < 0.05). Number of licks/session for ethanol was higher than that for water for all blocks (Newman-Keuls test, *P*’s < 0.001; A in S1 Fig).

Analysis of the ethanol preference data indicated a significant early increase in preference for 20% ethanol across session blocks (effect of block: *F*_14,140_ = 18.53, *P* < 0.001). Ethanol preference increased from 59.8% during the first three sessions to 81.4% during sessions 7–9, and then stabilized (Newman-Keuls test: block 1<2–15, block 2<3–15, *P*’s < 0.001; Fig 1C). Analysis of ethanol preference from the lick count data revealed a similar pattern to that of the fluid intake data, with slightly higher preference values (effect of block: *F*_14,140_ = 7.00, *P* < 0.001; Newman-Keuls test: blocks 1<2–15, block 2<3–15, *P*’s < 0.05; mean preference: block 1–71.8%, block 3–85.7%; B in S1 Fig).

### Microstructural Measures of Ethanol Ingestion

The increased intake and preference in P rats during initial ethanol sessions was driven by an increase in both the size and duration of ethanol drinking bouts, as well as the rate of ethanol ingestion within bouts. A one-way repeated measures ANOVA on bout size revealed a significant effect of block (*F*_14,140_ = 6.31, *P* < 0.001), with the size of alcohol drinking bouts increasing early in the chronic exposure phase (Newman-Keuls test: block 1<2–15, block 2<12–15, *P*’s < 0.01; C in S1 Fig). Analysis of bout duration indicated a significant increase across session blocks 1–6 (effect of block: *F*_14,140_ = 6.41, *P* < 0.001; Newman-Keuls test: block 1<4–15, block 2<3–15, block 3<6, *P*’s < 0.05), followed by stabilization (D in S1 Fig). Within-bout rate of ethanol ingestion rose after the first session block, with small increases as well later in the chronic exposure period (effect of block: *F*_14,140_ = 7.12, *P* < 0.001; Newman-Keuls test: block 1<2–15, blocks 4–6<12,14–15, blocks 7–8<15, *P*’s < 0.05; E in S1 Fig). Frequency of drinking bouts for both ethanol and water in ethanol-exposed subjects declined across the first six session blocks (main effect of block: *F*_14,140_ = 18.93, *P* < 0.001, Newman-Keuls test: blocks 1–2>3–15, block 3>6–7,9,11,13–15, block 4>11, *P*’s < 0.05), but the number of drinking episodes for ethanol was consistently higher than that for water throughout the experiment (main effect of solution: *F*_1,10_ = 42.82, *P* < 0.001; F in S1 Fig).

### Overall Fluid Intake and Body Weight–Ethanol vs. Control Subjects

Analysis of overall lick counts in ethanol-exposed and control subjects on stimulus exposure days revealed a significant effect of block (*F*_14,266_ = 4.43, *P* < 0.001) and a treatment × block interaction (*F*_14,266_ = 3.40, *P* < 0.001). Overall total licks/session in ethanol-exposed P rats did not significantly differ across block (Newman-Keuls test: *P*’s > 0.60; mean lick counts: block 1–4268.83 (±235.57), block 15–4391.08 (±279.78)), while total licks/session in control subjects displayed a modest decline from early to later sessions (Newman-Keuls test: blocks 1,4–5>11–15, block 2>9–15, block 3>10–15, block 5>12–15, *P*’s < 0.05; mean lick counts: block 1–3698.07 (±430.26), block 15–2797.90 (±238.87)). Despite the latter, comparison of total lick counts between ethanol-exposed and control subjects at each session block indicated no significant differences in overall intake (*P*’s > 0.36). There were also no significant differences in body weight between ethanol and control rats at any time during the chronic exposure phase (main effect of day only: *F*_45,855_ = 480.32, *P* < 0.001; effect of treatment and treatment × day interaction *NS*: *F*’s ≤ 0.28, *P*’s > 0.96; mean body weight: Day 1: control–408.00 (±21.32) g, ethanol–411.00 (±17.43) g; Day 94: control–585.90 (±17.41) g, ethanol–586.36 (±11.72) g).

### Lipid Profiles

**Fig 2:**
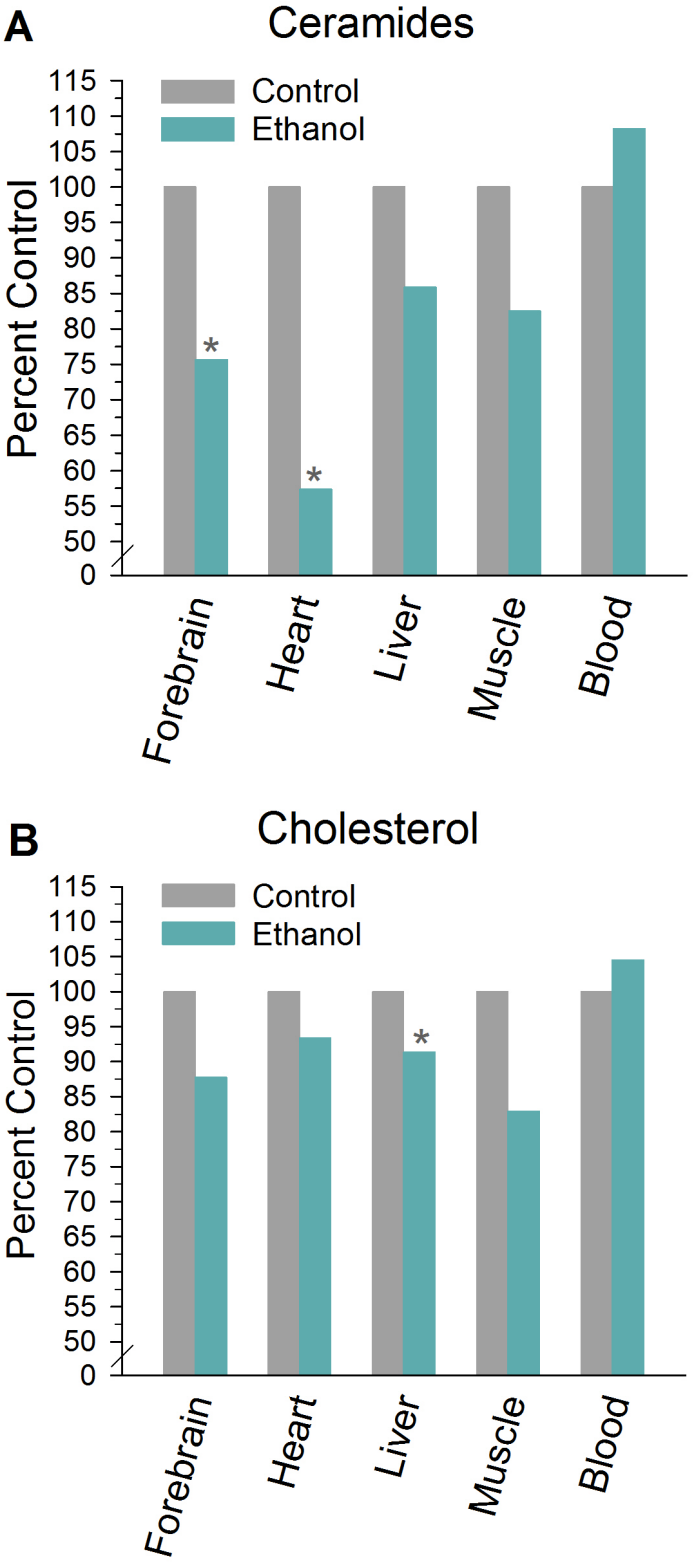
Levels of ceramides (A) and cholesterol (B) for each tissue type (represented as percent control) in selectively bred alcohol-preferring (P) rats exposed for 13 weeks to a 20% ethanol intermittent access drinking paradigm or given access to water alone (non-exposed control). Percent control = ((mean optical density value for ethanol/mean optical density value for control) × 100). *Significant difference between ethanol and control within tissue type (*P*<0.05).

One-way between-subjects treatment ANOVAs on the lipid optical density values revealed a significant reduction in levels of ceramides in ethanol-exposed P rats relative to non-exposed controls in both forebrain (24% reduction; effect of treatment: *F*_1,16_ = 12.34, *P* < 0.01) and heart (43% reduction; effect of treatment: *F*_1,18_ = 12.73, *P* < 0.01; Fig 2A). Mean (±SEM) optical density values for ethanol-exposed and control subjects are presented in Table 1. Ethanol-exposed animals also exhibited a modest but significant decrease in liver cholesterol (9% decline; effect of treatment: *F*_1,17_ = 6.72, *P* < 0.05) and a borderline decrease in forebrain cholesterol concentrations (*F*_1,16_ = 3.93, *P* = 0.06) compared to controls (Fig 2B). Additionally, levels of phosphatidylethanolamine (PE) in muscle were observed to be significantly lower in ethanol-treated P rats relative to non-exposed controls (effect of treatment: *F*_1,18_ = 7.67, *P* < 0.05), while blood sphingosine levels were elevated (effect of treatment: *F*_1,9_ = 24.50, *P* < 0.001). There were no significant differences in optical density values for phosphatidylcholine as a function of treatment (*F*’s ≤ 1.20, *P*’s > 0.31; Table 1). Forebrain sphingosine levels were undetectable using these chromatographic methods.

**Table 1:** Mean (±SEM) Optical Density Values by Lipid and Treatment Condition for Each Tissue Type.

	Forebrain	Heart	Liver	Muscle	Blood
**Ceramides**					
Control	7448.89^†^	3969.22^†^	4216.40	6183.50	8125.80
	(±375.90)	(±237.64)	(±776.83)	(±482.16)	(±479.01)
Ethanol	5634.22	2276.09	3619.40	5101.36	8795.50
	(±354.19)	(±381.41)	(±545.37)	(±639.12)	(±532.97)
**Cholesterol**					
Control	2705.22	1984.40	2460.22^†^	1877.43	2862.00
	(±126.01)	(±153.96)	(±75.28)	(±210.51)	(±103.86)
Ethanol	2375.11	1853.27	2248.50	1557.43	2992.00
	(±108.81)	(±147.42)	(±37.85)	(±171.16)	(±135.69)
**Phosphatidylcholine**					
Control	5152.40	4365.60	3418.80	2157.00	6523.60
	(±506.15)	(±582.85)	(±306.79)	(±371.83)	(±630.39)
Ethanol	5055.33	4517.46	3200.82	2077.55	7033.17
	(±845.85)	(±705.81)	(±487.03)	(±506.77)	(±352.74)
**Phosphatidylethanolamine**					
Control	3274.67	2814.10	2328.10	2830.70^†^	4432.80
	(±234.21)	(±167.83)	(±110.78)	(±352.70)	(±626.03)
Ethanol	2879.27	2693.80	2318.91	1763.00	5172.33
	(±453.17)	(±141.07)	(±146.36)	(±155.64)	(±458.94)
**Sphingosine**					
Control	——————-	2903.80	1527.70	239.00	217.60^†^
		(±584.78)	(±402.52)	(±62.45)	(±33.01)
Ethanol	——————-	2776.83	1025.09	190.50	466.33
		(±423.60)	(±249.70)	(±41.43)	(±36.60)

† indicates significant difference (*P* < 0.05) between ethanol and control within tissue type.

### Gene Expression Profiles

**Fig 3:**
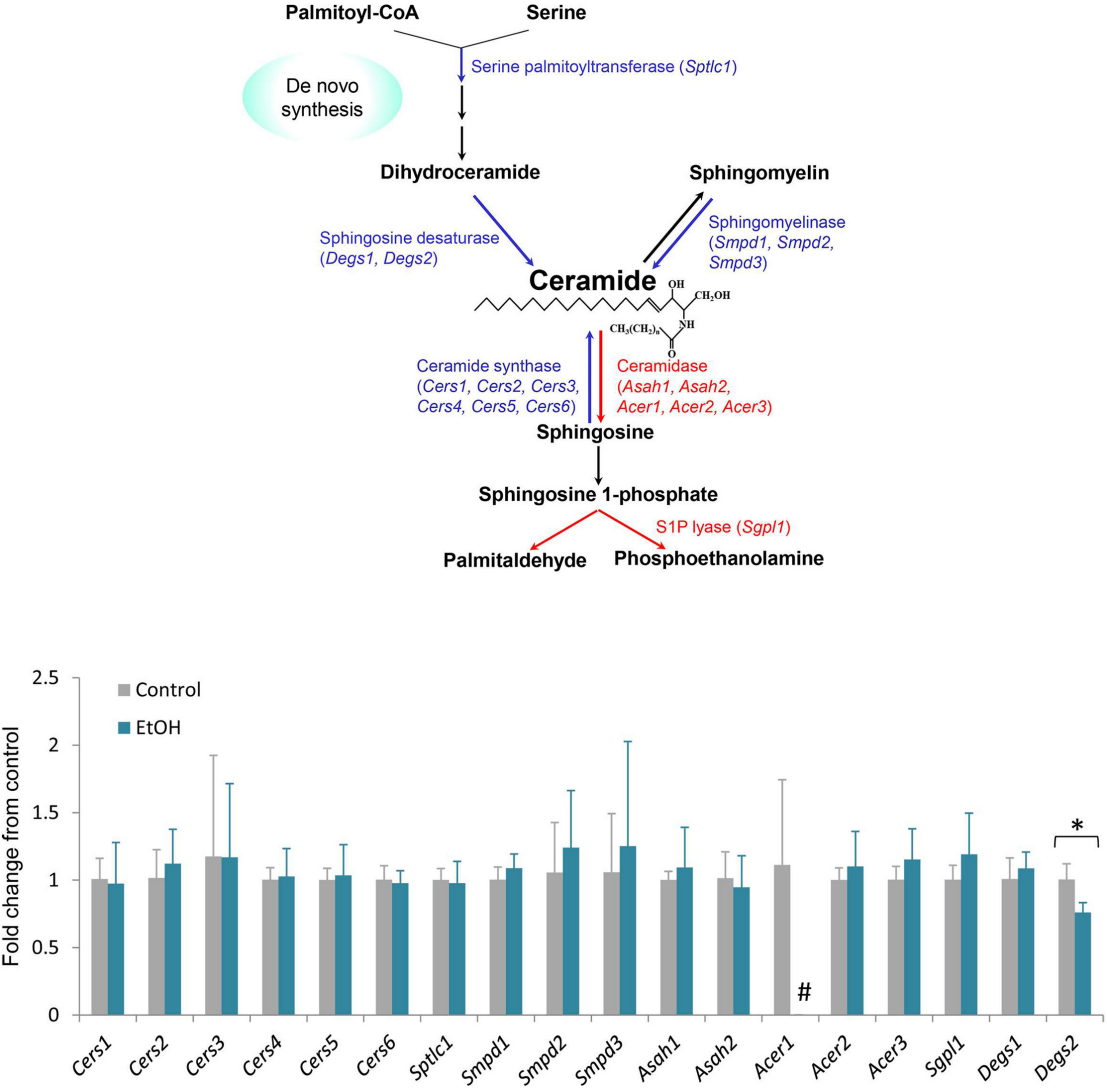
mRNA levels for genes encoding sphingolipid metabolic enzymes were determined in control and ethanol-exposed (EtOH) forebrain tissues by qRT-PCR. Expression was normalized to control rats. Error bars represent standard deviation. N = 4, *P<0.05, # = not detectable.

To determine whether the reduction of ceramide in ethanol-treated rats could be the result of alterations in gene expression, we performed qRT-PCR on the forebrain regions of control and ethanol-exposed rats. All known isoforms of ceramide synthase, ceramidase, sphingolipid desaturase, sphingomyelinase, and serine palmitoyl transferase were evaluated (Fig 3). Expression levels of these genes were largely unchanged, however, there was a moderate (24%) statistically significant decrease in a sphingolipid desaturase isoform, *Degs2*. In addition, alkaline ceramidase 1 (*Acer1*), which is expressed at relatively low levels in control rats, became undetectable after chronic ethanol exposure.

## Discussion

The present study measured the effect of chronic voluntary ethanol consumption on brain and peripheral lipid profiles in genetically selected adult alcohol-preferring (P) rats to further examine alterations in tissue lipid composition as a potential contributor to ethanol-induced pathology. Contrary to expectations, selectively bred alcohol-preferring (P) rats exposed chronically to a 20% ethanol intermittent access drinking paradigm displayed substantial reductions in concentrations of ceramides in both forebrain and heart relative to non-exposed controls, as well as modest but significant decreases in liver cholesterol. These data are opposite in nature to previously observed elevated levels of pro-apoptotic lipids, including ceramide, in developing rodents following acute ethanol administration, believed to contribute to fetal alcohol-induced neuronal loss [21–22]. The present findings indicate that ethanol intake levels achieved by alcohol-preferring P rats as a result of chronic voluntary exposure may have favorable vs. detrimental effects on lipid profiles in this genetic line, consistent with prior data supporting beneficial cardioprotective and neuroprotective effects of moderate ethanol consumption [3–4,32–38].

The intermittent access 20% ethanol drinking paradigm resulted in largely stable levels of ethanol intake and preference in alcohol-preferring P rats across the 13-week period of chronic exposure. Following an initial elevation in overall ethanol consumption due to an increase in both the size and duration of ethanol drinking episodes and within-bout rate of drinking, P rats subsequently consumed ~85% of their total fluid intake as 20% ethanol, and maintained average daily ethanol intake values of ~10 g/kg. These data are similar to previously reported intake levels (~8–9 g/kg/day final intake) by alcohol-preferring P rats in the 20% ethanol intermittent access paradigm [27–28]. Despite relatively high daily g/kg alcohol intake values achieved by P rats in this paradigm, measurement of blood alcohol levels (BALs) 30 min into a standard drinking session (when intake levels typically peak) have demonstrated only moderate levels of ethanol in blood in this genetic line, in the range of 11–63 mg/dl [27]. The latter blood alcohol levels are within a range corresponding to an acute 0.5 g/kg dose in human heavy drinkers [39], or that normally resulting from 1–2 standard drinks. These data indicate that in the present intermittent access paradigm, P rats may actually be maintaining more modest BALs than that expected based on absolute 24 hr g/kg intake values.

Analysis of tissue lipid profiles following the chronic exposure phase revealed significantly lower levels of ceramides in both heart and forebrain (43% and 24% decrease, respectively), and liver cholesterol (9% decline), in ethanol-exposed P rats compared to non-exposed controls. These data are the first we are aware of to report decreased ceramide concentrations as a result of chronic voluntary moderate ethanol exposure in adult organisms. In contrast to the present ethanol-induced reduction in ceramide concentrations, prior data in fetal models have demonstrated increased levels of ceramide in the brains of offspring following acute administration of a 1.5 g/kg dose during gestation [22] or a 5 g/kg cumulative dose on postnatal day 7 [21], approximating third trimester exposure in humans. The latter findings are also consistent with our own unpublished observations (Thomas, J.D. and Harris, G.L.) of elevated ceramide levels in multiple brain regions following ethanol exposure during a period of development equivalent to late gestation in humans. In those studies, pups were administered 5.25 g/kg/day in a binge-like manner using gastric intubation on postnatal days 4–9. Brain ceramide levels were measured on PD35, thus demonstrating a long-lasting elevation in brain bioactive lipid profiles. It is possible that age-related variables, as well as differing blood alcohol concentrations, may be significant contributing factors to the differential findings in ceramide profiles, as the acute ethanol doses administered by injection [21] or intubation [22] in the former studies would be expected to result in higher BACs (particularly in neonatal rodents) than those previously reported via voluntary drinking by adult P rats in the intermittent access paradigm (11–63 mg% [27]). It is also likely that genetic factors may play a role in ethanol-induced effects on lipid profiles, as significant differences in liver lipid content and associated alcohol-mediated neurodegeneration have previously been reported among different strains of adult rodents following chronic alcohol feeding via a liquid diet [20].

There are a number of biochemical processes that may lead to a reduction in ceramide, but these necessarily involve either a decrease in synthesis and/or an increase in degradation. To begin to address this mechanism, we examined the mRNA levels of genes that encode key enzymes in the ceramide metabolic pathway. We have shown in previous studies that genetic alterations of these enzymatic steps result in significant changes in ceramide accumulation [40]. Our results demonstrate here that most of the metabolic genes are unaltered by chronic ethanol exposure at the level of gene expression with two notable exceptions (Fig 3). *Degs2*, a sphingolipid desaturase, is reduced by 24% in the brain. Since this enzyme catalyzes the conversion of dihydroceramide to ceramide, it is possible that reduction of Degs2 activity may limit ceramide accumulation. Interestingly, ethanol exposure caused an apparent reduction in *Acer1*, which encodes a ceramide-degrading enzyme. However, *Acer1* mRNA level in control rats is near the threshold of detection in these samples (Ct value ~36), which indicates that *Acer1* expression is very low compared to that of the other 5 ceramidase isotypes, and is unlikely to make a significant contribution to ceramide homeostasis in the forebrain. Therefore, we conclude that a reduction in ceramide synthesis may contribute to the lower ceramide levels observed. It will be of interest in future studies to evaluate levels of enzyme protein expression and their activities in order to characterize these changes further.

Prior epidemiologic data have established an association between moderate ethanol consumption and beneficial cardioprotective and neuroprotective effects, including a reduced risk for heart disease, myocardial infarction, cerebrovascular (ischemic) stroke, and decreased risk of age-related cognitive decline or dementia [3–4,33–38]. The relationship between ethanol consumption and adverse cardiovascular and neurological outcomes exhibits a J-shaped or U-shaped curve, with light to moderate drinking associated with decreased risk compared to abstinence, while high levels of alcohol consumption confer increased risk [3,34,38]. Experimental studies utilizing in vivo animal models and cardiac and brain cell culture preparations have also causally demonstrated that moderate ethanol exposure induces protection from ischemia-induced myocardial and neuronal injury [41–43], as well as neurotoxic damage induced by ß-amyloid or other pro-inflammatory agents (e.g., HIV-1 glycoprotein 120, lipopolysaccharide [44–47]). With regard to proposed mechanisms underlying these protective effects, clinical studies have provided evidence that moderate alcohol intake is associated with a reduction in plasma lipids, low-density lipoprotein (LDL) cholesterol and inflammatory cytokines, decreased platelet aggregration, increased levels of high-density lipoprotein (HDL) cholesterol, and enhanced insulin sensitivity [3,33,48–49]. Findings from rodent models involving chronic moderate alcohol feeding indicate that such exposure protects cardiac myocytes directly against subsequent ischemic injury via sustained activation of mitochondrial protein kinase C episilon [41], a powerful cytoprotectant [50], as well as improves the functioning of the vascular endothelium via increased expression of endothelial nitric oxide synthase (eNOS [51]), resulting in improved blood flow and blood pressure. Collectively, existing data indicate that moderate ethanol exposure engages anti-inflammatory processes in the heart, vasculature, and CNS that promote cytoprotection and cell survival [3,34].

The present findings of ethanol-induced reductions in heart and forebrain ceramide levels provide novel data that may be mechanistically related to the cardioprotective and neuroprotective effects of moderate ethanol exposure. Ceramide homeostasis has been shown to be critical in cardiac function, with excess ceramide accumulation implicated in the pathogenesis of lipotoxic cardiomyopathy [52–53] as well as myocardial ischemia/reperfusion injury [54] in rodent models. There is also an association between plasma ceramide content and levels of inflammatory markers related to insulin resistance in patients with coronary heart disease [55] and risk factors for atherosclerosis (e.g., increased total cholesterol and triglycerides [56]). Conversely, pharmacological inhibition of ceramide synthesis has been shown to directly improve insulin resistance, including cardiac glucose utilization, in models of diet-induced obesity [57–59]. Decreased cardiac accumulation of ceramides as a result of ischemic preconditioning has also been proposed in part to underlie the protective effects of this procedure against myocardial ischemia/reperfusion damage [54]. Interestingly, the protective effects of antecedent moderate alcohol exposure in reducing I/R injury have been shown to closely resemble the effects of ischemic preconditioning [3,34]. With regard to central nervous system effects, elevated levels of ceramides have also been implicated in the cell death accompanying a variety of neuroinflammatory and neurodegenerative disorders, including Alzheimer’s disease, HIV-associated dementia, multiple sclerosis, ALS, and stroke ([15], for review), as well as alcohol-induced neuronal damage [19–21]. Such data would be consistent with reduction in brain ceramide levels potentially inducing beneficial neuroprotective effects. Relevant to the current study, one prior report in alcohol-preferring (P) rats exposed chronically to 15% ethanol via voluntary drinking demonstrated that cortical neurons isolated from these animals were protected from apoptosis induced by prior injection of pro-inflammatory lipopolysaccharide [46].

Importantly, the reduction in ceramide levels observed in the current study, as well as previously reported protective effects of ethanol exposure, have been observed in protocols yielding blood alcohol levels generally in a range of 10–67 mg/dl [27,60–64]. A significant literature supports detrimental health effects [4,36] and elevations in ceramide concentrations [21,65] at higher levels of ethanol exposure. A recent study by Roux and colleagues (2015) utilizing a binge-like ‘drinking-in-the-dark’ ethanol exposure paradigm in adult C57BL/6 mice, which typically results in blood ethanol levels in excess of 100 mg% [66–69], reported an elevation in ceramide content in various brain regions when tissues were collected 1 hr post-ethanol access [65]. The more moderate BALs achieved by P rats in the 20% ethanol intermittent access paradigm [27] indicates that these subjects may be distributing their intake over time and thus maintaining more modest BALs on a chronic basis, a potentially critical variable in the differential observed ceramide profiles resulting from chronic consumption. It is further important to note that the reduction in ceramide levels observed here following chronic moderate ethanol exposure reflect sober levels (i.e., tissues collected the day after the last ethanol session), at a time when animals were not intoxicated and ethanol was absent from blood. A recent report in adult C3-C57BL/6J mice demonstrated that short-term exposure in a binge-like model (5 days of access to 20% ethanol for 3 hr/day) led to decreased brain ceramide concentrations during a state of acute intoxication, but elevated levels during acute withdrawal (6 hr post-ethanol) [70]. These data reinforce that the time point at which ceramide levels are assessed relative to ethanol exposure (i.e., acute intoxication, immediate withdrawal, or protracted abstinence) is an important variable for further examination that can produce unique physiological profiles. Immediate levels of ethanol in blood, as well as levels maintained over chronic exposure, may also influence potential neuroprotective or neurotoxic effects resulting from alterations in ceramide phenotype. It would be very interesting for subsequent studies to determine the long-term persistence of the observed reduction in cardiac and forebrain ceramide concentrations as a consequence of chronic moderate ethanol consumption, as well as potential region-specific decreases in forebrain ceramide levels resulting from such exposure, particularly given prior data that light-to-moderate drinking has been associated with reduced risk for dementia and cardiovascular disease [3–4].

In summary, the present data indicate that a chronic alcohol exposure paradigm resulting in moderate blood alcohol levels in adult alcohol-preferring (P) rats produces favorable effects on heart and brain ceramide profiles. Future experiments should systematically examine alcohol dose-related effects on tissue ceramide concentrations and related functional outcomes, the influence of age and genetic (e.g., strain-related) factors on these measures, and how timing relative to ethanol exposure (i.e., acute intoxication, withdrawal, and long-term abstinence) differentially influences ceramide and other lipid levels. While not diminishing the damaging effects of heavy alcohol consumption, these findings add to a growing literature that moderate ethanol intake has beneficial effects on specific physiological and behavioral indices of disease risk, and may contribute to a more thorough understanding of the underlying mechanisms mediating ethanol’s cardioprotective and neuroprotective effects.

## Supporting Information

S1 Fig Mean (±SEM) total licks/session to ethanol and water (A), percent preference for ethanol ((total licks to ethanol/total licks to ethanol and water) × 100; B), size (# licks/bout) and duration (min) of ethanol drinking bouts (C,D), within-bout rate of ethanol ingestion (# licks/sec; E), and frequency of ethanol and water drinking bouts (F) in selectively bred alcohol-preferring (P) rats across all 15 session blocks of ethanol exposure (45 sessions total) in an intermittent-access 20% ethanol drinking paradigm. A drinking episode or “bout” was operationally defined as an occurrence of 20 or more successive licks on a given tube separated by interlick intervals of less than two minutes. Individual session data were averaged in 3-session blocks prior to analysis.
(PDF)

S1 Appendix Gene abbreviations and full name equivalents for gene expression analysis. (PDF)

S2 Appendix The Animal Research: Reporting In Vivo Experiments (ARRIVE) Guidelines Checklist. (PDF)
