# Supplementary figures and images for: Chronic Voluntary Ethanol Consumption Induces Favorable Ceramide Profiles in Selectively Bred Alcohol-Preferring (P) Rats
Source: PLoS One. 2015 Sep 25;10(9):e0139012. doi: 10.1371/journal.pone.0139012 (PMC4583526; doi:10.1371/journal.pone.0139012)

S1 Fig

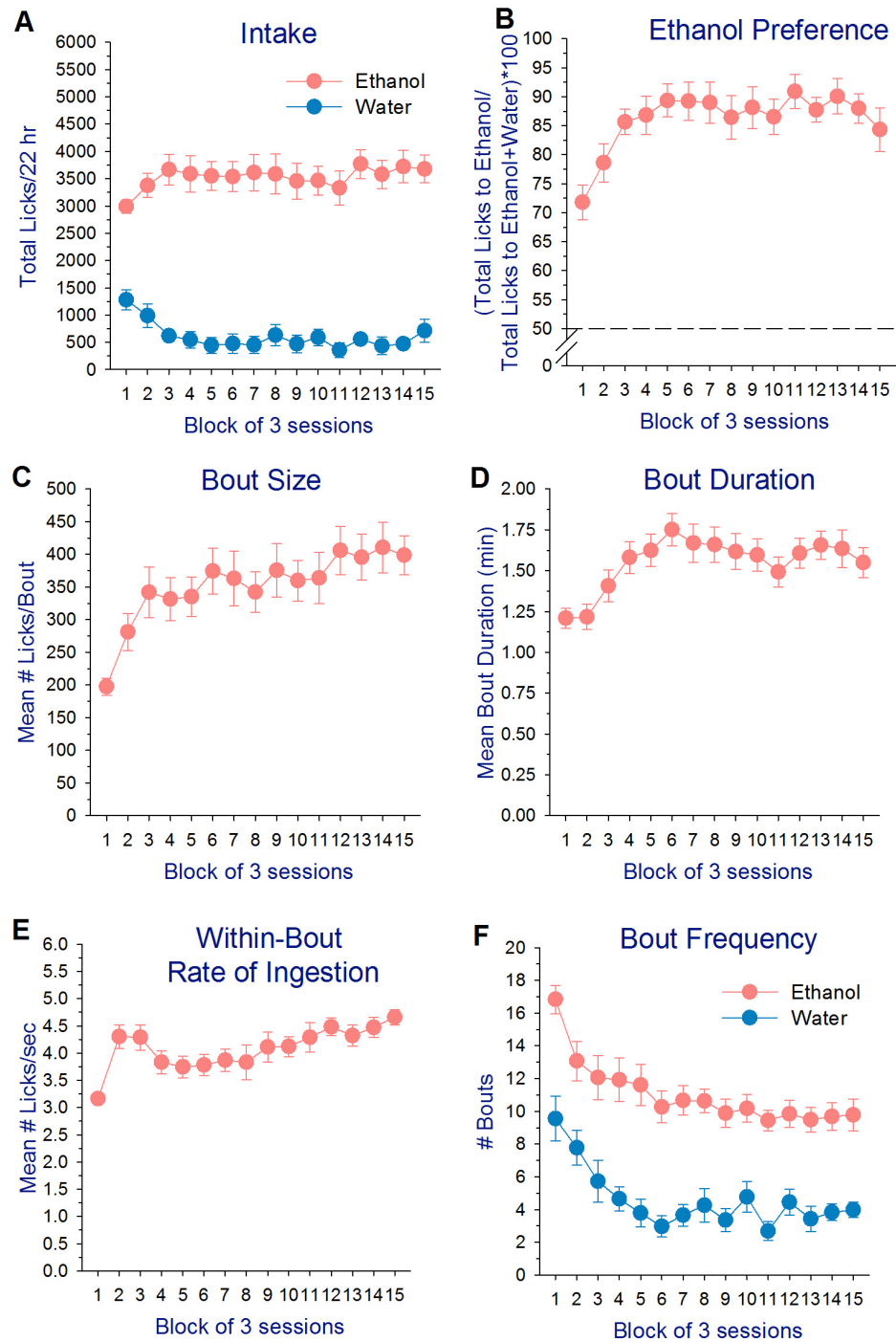

Supplement: S1 Fig — A drinking episode or “bout” was operationally defined as an occurrence of 20 or more successive licks on a given tube separated by interlick intervals of less than two minutes. Individual session data were averaged in 3-session blocks prior to analysis. (PDF) [file pone.0139012.s001.pdf]
