# Supplementary material for: Chronic Voluntary Ethanol Consumption Induces Favorable Ceramide Profiles in Selectively Bred Alcohol-Preferring (P) Rats
Source: PLoS One. 2015 Sep 25;10(9):e0139012. doi: 10.1371/journal.pone.0139012 (PMC4583526; doi:10.1371/journal.pone.0139012)
Supplement: S1 Appendix — (PDF) [file pone.0139012.s002.pdf]

S1 Appendix

Sptlc1 - serine palymitoyltransferase

Cers1-6 - ceramide synthase

Smpd1-3 - sphingomyelinase

Asah1-2 - acid ceramidase

Acer1-3 - alkaline ceramidase

Sgpl1 - S1P lyase
